# Use and overuse of proton pump inhibitors

**DOI:** 10.1186/1824-7288-41-S2-A68

**Published:** 2015-09-30

**Authors:** Annamaria Staiano, Paolo Quitadamo

**Affiliations:** 1 Department of Translational Medical Science, Section of Pediatrics, University of Naples “Federico II”, Naples, Italy

## 

**Figure 1:**
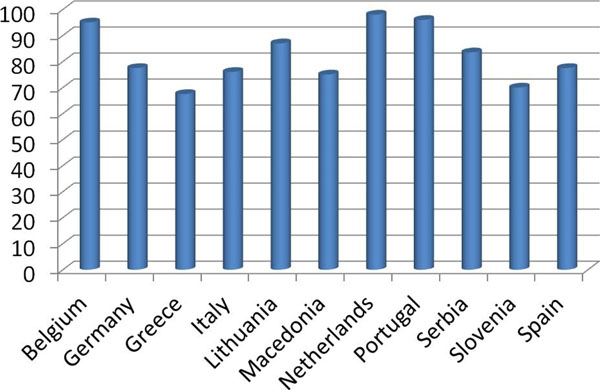
Rates of pediatricians over-prescribing PPIs in the different European countries

In 2009, an editorial by Putman focused on the dramatic increase in the use of proton pump inhibitors (PPIs) in infants [1]. Considering the increasing evidence that PPIs offer little benefit for most of the symptoms for which they are prescribed, Putman strongly highlighted the need for a serious effort to curtail their empiric use. The editorial was linked to a study by *Orenstein et al*, which detected no difference in efficacy between lansoprazole and placebo for symptoms attributed to gastroesophageal reflux disease (GERD) in infants [2]. A very recent review on the effects of PPIs on irritability and crying in infants confirmed the conclusion that these drugs do not offer relief from these symptoms for which they are commonly prescribed [3]. After all, it has recently been pointed out that volume rather than acid is often the key promoter of reflux-associated symptoms in infants. Distension-induced increase in gastric wall tension has been associated with high rates of transient lower esophageal sphincter relaxations and is considered one of the main mechanisms contributing to postprandial GER [4]. Despite all these evidences on PPIs uneffectiveness for many of the symptoms for which they are commonly prescribed, the rate of their prescriptions have exponentially increased over the last years. The latest combined NASPGHAN/ESPGHAN guidelines already in 2009 highlighted the need to distinguish physiologic GER events from GERD, limiting for the latter the use of PPIs [5]. However, a 2012 European survey carried out by our study group showed that only 1.8% of pediatricians reported a complete adherence to the guideline recommendations in their clinical practice. The most relevant violation of the guideline recommendations concerned the prescription of unnecessary acid suppressive medications [6] (Figure 1). In order to inquire into the reasons why pediatricians did not follow the guideline recommendations, we performed another trial with the same pediatricians undergoing specific training on the guidelines being supervised in their clinical practice. As a result, trained pediatricians showed a significantly higher compliance with the international guidelines and infants with unexplained crying and distressed behaviour or with uncomplicated recurrent regurgitation and vomiting who were unproperly prescribed PPIs decreased dramatically [7]. Besides these data on PPI overuse, recent studies highlighted the occurrence of several adverse effects related to their assumption, such as a higher risk of bronchoalveolar lavage culture positivity and gastric bacterial overgrowth, food allergy, *Clostridium difficile* infection, gastric polyps and nodules [8-12].
